# Physiological levels of lipoxin A_4_ inhibit ENaC and restore airway surface liquid height in cystic fibrosis bronchial epithelium

**DOI:** 10.14814/phy2.12093

**Published:** 2014-08-08

**Authors:** Mazen Al‐Alawi, Paul Buchanan, Valia Verriere, Gerard Higgins, Olive McCabe, Richard W. Costello, Paul McNally, Valérie Urbach, Brian J. Harvey

**Affiliations:** 1 Department of Molecular Medicine, Royal College of Surgeons in Ireland, Education and Research Centre, Beaumont Hospital, Dublin 9, Ireland; 2 Department of Respiratory Medicine, Royal College of Surgeons in Ireland, Education and Research Centre, Beaumont Hospital, Dublin 9, Ireland; 3 National Children Research Centre, Dublin 12, Ireland; 4 INSERM U661, Montpellier, France

**Keywords:** Cystic fibrosis, ENaC, lipoxin A_4_

## Abstract

In cystic fibrosis (CF), the airway surface liquid (ASL) is depleted. We previously demonstrated that lipoxin A_4_ (LXA_4_) can modulate ASL height (ASLh) through actions on Cl^−^ transport. Here, we report novel effects of lipoxin on the epithelial Na^+^ channel ENaC in this response. ASL dynamics and ion transport were studied using live‐cell confocal microscopy and short‐circuit current measurements in CF (CuFi‐1) and non‐CF (NuLi‐1) cell cultures. Low physiological concentrations of LXA4 in the picomolar range produced an increase in ASLh which was dependent on inhibition of an amiloride‐sensitive Na^+^ current and stimulation of a bumetanide‐sensitive Cl^−^ current. These ion transport and ASLh responses to LXA_4_ were blocked by Boc‐2 an inhibitor of the specific LXA4 receptor ALX/FPR2. LXA_4_ affected the subcellular localization of its receptor and enhanced the localization of ALX/FPR2 at the apical membrane of CF cells. Our results provide evidence for a novel effect of low physiological concentrations of LXA_4_ to inhibit airway epithelial Na^+^ absorption that results in an ASL height increase in CF airway epithelia.

## Abstract (precis)

LXA4 induced apical membrane ALX/FPR2 localization in CuFi‐1 monolayers. LXA4 induced an apical increase of ALX/FPR2 (green) expression (B). Primary rabbit anti‐ALX/FPR2 antibody and secondary Alexa‐Fluor 488 anti‐rabbit were used to label the ALX/FPR2 receptor. Localization of the receptor at the apical surface is shown in the merged fluorochrome images in yellow (A). Rhodamine‐phalloidin was used to stain f‐actin and DAPI used to stain the nuclei (C).

## Introduction

The surface of the bronchi is covered by an airway surface liquid (ASL) layer which is maintained at optimum height by NaCl transport across the epithelium. Sodium absorption through the epithelial Na^+^ channel (ENaC) induces water absorption and dehydrates the ASL, whereas secretion of Cl^−^ via the cystic fibrosis transmembrane conductance regulator (CFTR) and Ca^2+^‐activated Cl^−^ channels have the opposite effect to hydrate the ASL (Boucher 2004b; Blouquit‐Laye and Chinet 2007).

Cystic fibrosis (CF) is a lethal genetic disorder resulting from a mutation of the CFTR gene (Riordan et al. 1989). CFTR functions as a Cl^−^ channel and as a regulator of other ion channels. In CF, the absence of CFTR inhibition of ENaC results in increased isotonic absorption of Na^+^ and dehydration of the ASL layer (Stutts et al. 1995; Kunzelmann et al. 1997). ASL dehydration results in chronic bacterial infection, persistent inflammation and progressive lung destruction. The greatest therapeutic challenge in CF is to restore ion transport function, enhance ASL dynamics, and reduce infection (Tarran et al. 2001; Pisi and Chetta 2009).

The endogenous lipoxin A_4_ is an eicosanoid that triggers resolution of inflammation in a wide variety of tissues. Lipoxins have been proposed as novel regulators of immunity and may have therapeutic potential in chronic immune disorders (Serhan et al. 1984a). LXA_4_ was shown to decrease pro‐inflammatory cytokine IL‐8 release, arrest neutrophilic inflammation, and decrease infection in a mouse model of chronic airway inflammation and infection (Karp et al. 2004). CF has been associated with a reduced LXA_4_ level in bronchoalveolar lavage (Karp et al. 2004; Starosta et al. 2006).

We have previously shown that high concentrations of LXA_4_ (100 nmol/L) stimulated airway epithelium tight junction formation (Grumbach et al. 2009), produced an intracellular Ca^2+^ mobilization and Cl^−^ secretion (Bonnans et al. 2003), and enhanced ASL height in bronchial epithelial cells derived from patients with CF (Verriere et al. 2012). Here, we report a role for low physiological concentrations of LXA_4_ (1 nmol/L) in correcting Na^+^ and Cl^−^ transport and ASL height in a human cell model of CF airway epithelium. These studies suggest a therapeutic role for lipoxins as novel regulators for the correction of Na^+^ and Cl^−^ transport dysfunction in CF airway.

## Methods

### Cell culture and Ussing chamber experiments

NuLi‐1 and CuFi‐1 cell lines donated by Prof Zabner (University of Iowa, USA) have typical bronchial epithelial phenotype of mucus secretion and air–surface liquid generation. The NuLi‐1 cell line was derived from human airway epithelium of normal genotype, whereas the CuFi‐1 line was derived from a CF patient with Δ508/Δ508 genotype (Zabner et al. 2003). NuLi‐1 and CuFi‐1 epithelia were grown to well‐differentiated monolayers under an air–liquid interface (ALI). Cells were initially grown to confluency in flasks using bronchial epithelial growth medium (BEBM; Lonza, Bethesda, MD) with epidermal growth factor, hydrocortisone, bovine pituitary extract, transferring, bovine insulin, tri‐iodothyronine, epinephrine, retinoic acid, penicillin‐streptomycin (0.025 *μ*g/mL), gentamicin (0.05 ng/mL), and amphotericin (25 *μ*g/mL). When cell confluence was confirmed under visual inspection, the medium was switched to DMEM/F‐12 (Invitrogen, Auckland, New Zealand) to aid cell differentiation. This medium was supplemented with Ultroser G (2%, Pall Biospera, Cergy‐Saint‐Christophe, France), which enhances ion transport (Zabner et al. 2003), and penicillin‐streptomycin (0.025 *μ*g/mL), gentamicin (0.05 ng/mL), and amphotericin (25 *μ*g/mL). The culture medium at the apical aspect was aspirated every 3–4 days until the establishment of an air–liquid interface. The basolateral culture medium was replaced every 2–3 days. After 4–6 weeks, the cells formed a polarized confluent monolayer, differentiated clear cells, and mucin‐secreting cells, beating cilia and a high transepithelial electrical resistance (TER) of >700 Ω/cm^2^.

### Airway surface liquid height measurements

The ASL height in Nuli‐1 and CuFi‐1 epithelia was measured by confocal fluorescence microscopy using a protocol adapted from (Tarran and Boucher 2002). To label the ASL, 8 *μ*L PBS containing 1 mg/mL Texas red^®^‐dextran (10 kD; Invitrogen) was added to the apical fluid overlying the well‐differentiated airway epithelium. The epithelial cells were stained using calcein green‐AM (5 µmol/L, Invitrogen) dissolved in medium culture for 30 min and introduced to the basolateral compartment of the insert. The Fluorinet^™^ electronic fluid Perfluorocarbon 72 (FC‐72; 3M, St Paul, MN) was added to the apical compartment of the insert at a volume of 0.5 mL. Perfluorocarbon 72 is immiscible with the ASL and was used to prevent ASL evaporation on transferring the inserts from the incubator to the microscope stage and during the confocal scanning experiments. Fluorescent images of the epithelial layer and ASL height were obtained using a confocal microscope (Zeiss LSM 510 Meta 40× objective, Jena, Germany). The average ASL height was recorded within the microscope field from a XZ scan of a 9‐point square matrix yielding eight separate ASL height measurement vectors. Images were analyzed using the Zeiss LSM Image analyzer software (Carl Zeiss Microlmaging GmbH, Jena, Germany).

### Immunocytochemistry

NuLi‐1 and CuFi‐1 cells were grown in air–liquid interface until differentiation at 4–6 weeks and finally fixed in 4% (w/v) paraformaldehyde in PBS for 30 min on ice. After two washes in PBS, cells were then permeabilized in 0.4% (v/v) Triton‐X‐TBS for 5 min, and then blocked in 3% (w/v) BSA in PBS. Cells were incubated for 2 h on ice with the primary antibody,

To visualize the LXA_4_ receptor, a rabbit polyclonal anti‐FPRL‐1 antibody was diluted 1:600 in PBS. After three washes in PBS, cells were incubated for 1 h on ice with the secondary antibody; AlexaFluor 488‐conjugated anti‐Rabbit (Invitrogen) diluted 1:400 in 2% (w/v) PBS. Cells were washed three times in PBS and finally mounted in Vectashield mounting solution (Vector Laboratories, Burlingame, CA) containing DAPI blue.

The subcellular distribution of FPR2/ALXR was observed using a LSM 710 confocal microscope (Zeiss, Welwyn Garden City, UK), equipped with an Argon laser and a HeNe laser. The Alexafluor 488‐labeled anti‐rabbit antibody was visualized using a 488 nm excitation wavelength and 505–530 nm emission range. DAPI was visualized using a 364 nm excitation wavelength and 385–470 nm detection range. Rhodamine‐phalloidin was used to stain f‐actin filaments in the cellular cytoskeleton and was visualized using a 543 nm excitation wavelength and 560–630 nm detection range. To stain the plasma membrane, Wheat germ agglutinin (WGA) was used to visualize using a 568 nm excitation wavelength to highlight the plasma membrane.

### Short‐circuit current (I_SC_) recordings

Differentiated NuLi‐1 and CuFi‐1 epithelial monolayers were mounted in Ussing chambers (Physiological Instruments, San Diego, CA). The bathing Krebs solution was composed of 140 mmol/L NaCl, 5.2 mmol/L KCl, 0.8 mmol/L MgCl_2_, 1.2 mmol/L CaCl_2_, 0.4 mmol/L KH_2_PO_4_, 2.4 mmol/L K_2_HPO_4_, 25 mmol/L NaHCO_3_, 10 mmol/L HEPES (free acid), and 10 mmol/L Glucose. Chambers were constantly gassed with a mixture of 95%O_2_/5%CO_2_ at 37°C, which maintained the pH at 7.4 and established a circulating perfusion within the Ussing chamber. The spontaneous transmembrane potential was measured and clamped to 0 mV by application of a short‐circuit current using a voltage clamp model amplifier (EVC 4000, World Precision Instrument, Sarasota, FL).

### Flow cytometry

Cells, 5 × 10^5^, from ALI cultures were isolated and resuspended in 100 µL of FACS buffer (0.2% FCS, 0.02% sodium azide in PBS). A directly labeled IgG anti‐ALX/FPR2 antibody was added according to the manufacturers recommendations, followed by an IgG2b isotype control and incubated for 30 min on ice. Cells were then washed three times in cold PBS before suspension in 300 *μ*L FACS buffer. The samples were analyzed on an Epics XL‐MCL flow cytometer in triplicates (Beckman Coulter AB45298 using Expo 32 analysis (Applied cytometry systems, Sheffield, U.K.).

### Drugs

Lipoxin A_4_ was obtained from Calbiochem (Merck KGaA, Darmstad, Germany) and aliquots (10^−4^ mol/L in ethanol) were stored at −80°C. Where indicated, cells were preincubated with the peptide Boc‐Phe‐Leu‐Phe‐Leu‐Phe (Boc‐2) (Phoenix pharmaceutical, Burlingame, CA) at 10^−5^ mol/L for 60 min at 37°C before treatment with LXA_4_. Bumetanide (Sigma, St Louis, MO) was stored at 0.1 mol/L in DMSO and used at final concentration of 10 *μ*mol/L. Amiloride (1 *μ*mol/L; Sigma) applied on the apical side was dissolved in FC‐72.

### Statistical analysis

All the experiments were performed at least on three separate occasions (*n *≥ 3). Results are given as mean value ± standard error of the mean obtained from *n* independent experiments. Student's *t*‐test was used to compare two populations. Analysis of variance (ANOVA) was performed for multiple comparisons, *P* < 0.05 was treated as significant (*P < 0.05, **P < 0.01, ***P < 0.001).

## Results

### Steady‐state air–surface liquid dynamics

**Figure 1.**
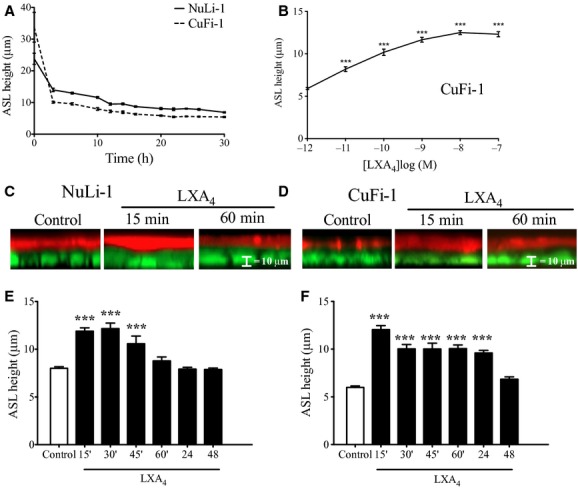
LXA_4_ pretreatment increases ASL height in NuLi‐1 and CuFi‐1 monolayers. (A) ASL height was measured over a period of 30 h to identify the time at which the ASL had stabilized. (B) LXA_4_ dose response (15 min treatment) on the ASL height increase in CuFi‐1 epithelium. (C) Typical confocal Z‐sections of Nuli‐1 and CuFi‐1 (D) cell monolayers obtained by confocal microscopy in control conditions and after 15‐ and 60‐min treatment by LXA_4_ (1 nmol/L). The ASL was stained with Dextran‐Texas Red and cell monolayer (stained green with Calcein‐AM). The effect of LXA_4_ (1 nmol/L) on the ASL height depth in NuLi‐1 nontreated epithelium and after 15, 30, 45, 60, min, 24 and 48‐h treatments (E). (F) The effect of LXA_4_ (1 nmol/L) on the ASL height measured 24 h after ASL labeling in CuFi‐1 in nontreated epithelium and after 15, 30, 45, 60 min, 24, and 48‐h treatments (***P < 0.001).

Airway Surface Liquid height (ASLh) measurements were carried out over a period of 30 h to follow the dynamics of epithelial fluid absorption/secretion and to determine the time course of the generation of a steady‐state thin ASL film in CuFi‐1 and NuLi‐1 cell monolayers cultured under an air–liquid interface. The rate of change in ASL height and the final steady‐state ASLh were compared between NuLi‐1 and CuFi‐1 monolayers following apical addition of a thin film of PBS solution to the epithelium to establish an airway surface liquid layer (Fig. 1A). The spontaneous change in ASL height reflects the combined effects of absorption and secretion of electrolyte and fluid on ASL volume. We observed a faster instantaneous decline in ASLh to a lower steady‐state level in CF compared to the non‐CF epithelia. At time zero of PBS addition, the “starting” ASLh was higher in CuFi‐1 compared to NuLi‐1 epithelia, which most probably reflects differences in the Texas red‐dextran diffusion within the mucus layers of different rheology between Nuli‐1 and CuFi‐1, before transepithelial ion transport starts to modify the ASL height. However, what is important for understanding the contribution of ion transport to ASLh generation is the rate of ASLh decline and the steady‐state equilibrium values. Following addition of PBS, NuLi‐1 monolayers exhibited a slower decline in ASLh (1.19 ± 0.02 *μ*m/h), which reached a steady‐state value of 7.97 ± 0.21 *μ*m (*n* = 27) after 12 h and 8.8 ± 0.32 *μ*m (*n* = 50) at 24 h. In contrast, CuFi‐1 epithelia exhibited a faster rate of decline in ASLh (2.24 ± 0.02 *μ*m/h) and a lower steady‐state ASLh of 5.7 ± 0.14 *μ*m (*n* = 36 epithelia) after 12 h and 6.0 ± 0.33 *μ*m (*n* = 81) after 24 h. The difference between the steady‐state ASLh in NuLi‐1 and CuFi‐1 was statistically significant across all time points measured (*P* < 0.001).

### Lipoxin A_4_ increases ASL height in CF and non‐CF epithelia

LXA_4_ exposure resulted in a rapid increase in ASLh within 15 min in both CuFi‐1 and NuLi‐1 epithelia. The LXA_4_ concentration dependence of this response was investigated in CuFi‐1 cells over a range 10^−12^ mol/L to 10^−7^ mol/L. The minimum LXA_4_ concentration that produced a significant ASLh increase was 10^−11^ mol/L and the maximum effect was obtained at 10^−8 ^mol/L (Fig. 1B).

The time dependence of the effect of LXA_4_ on ASLh was tested at 15‐, 30‐, 45‐, and 60‐min intervals following stabilization of the ASLh. LXA_4_ was applied for “short‐term” periods of 15, 30, 45, and 60 min in NuLi‐1 (Fig. 1C) and CuFi‐1 epithelia (Fig. 1D). LXA_4_ (1 nmol/L) treatment for 15 min increased ASLh by 51% in Nuli‐1 epithelia (*n* = 18) and doubled the ASLh in CuFi‐1 epithelia (*n* = 19). Longer LXA_4_ treatments did not produce any further stimulation of ASLh, which showed a slow decrease toward the steady state (Fig. 1C). Longer periods of LXA_4_ treatment for 24 h resulted in a sustained higher ASL in CuFi‐1 (Fig. 1D) but not in NuLi‐1 monolayers (Fig. 1C), and at 48 h the ASL height did not demonstrate a significant increase in either CuFi‐1 or NuLi‐1 monolayers.

Our experiments highlight that in control conditions, the non‐CF NuLi‐1 cell monolayers are overlaid with a continuous ASL layer, whereas the CF CuFi‐1 monolayers have a disrupted and thinner ASL layer. The zones of disrupted ASL could be explained by the presence of secreted mucins and a localized dehydration of the ASL. After LXA_4_ treatment of the CF cells, the ASL height increased and the disruption of the ASL was eliminated suggesting that LXA_4_ enhances fluid secretion into the ASL and inhibits mucin secretion by CF airway epithelial cells.

### The ALX/FPR2 receptor mediates lipoxin A_4_ effects on ASL height

Evidence supporting a role for the ALX/FPR2 receptor in mediating the effects of LXA_4_ came from studies using Boc‐2, a peptide antagonist first reported to block inflammation by binding to ALX/FPR2 (Gavins et al. 2003). The receptor recognizes a variety of peptides, synthetic or endogenously generated but with lower affinity compared to LXA_4_.

**Figure 2.**
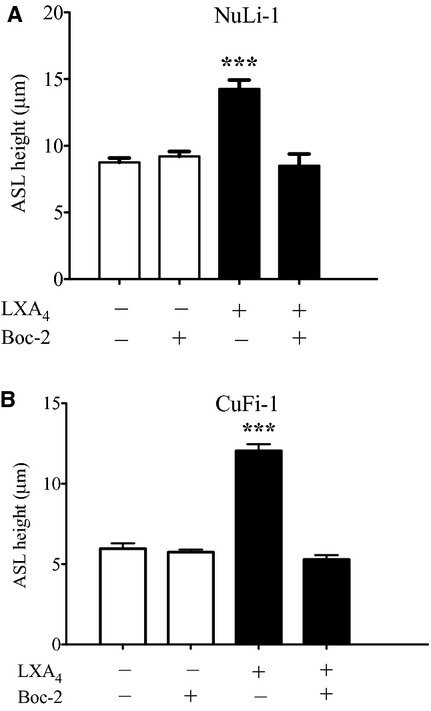
Boc‐2 attenuates the LXA_4_‐induced increase in ASL. Effect of Boc‐2 pretreatment to attenuate the LXA_4_ (1 nmol/L, 15‐min treatment) induced ASL height increase in NuLi‐1 (A) and CuFi‐1 (B) epithelial monolayers (***P < 0.001).

In NuLi‐1 cells, Boc‐2 pretreatment attenuated the LXA_4_ induced ASLh increase from 14.26 ± 0.67 *μ*m (*n* = 5) to 8.5 ± 0.88 *μ*m (*n* = 10, *P* < 0.001) (Fig. 2A). Similarly, in CuFi‐1 monolayers, Boc‐2 pretreatment inhibited the LXA_4_ stimulated ASLh increase from 12.04 ± 0.42 *μ*m (*n* = 8) to 5.29 ± 0.27 *μ*m (*n* = 3, *P* < 0.001) (Fig. 2B).

### LXA_4_ increases apical ALX/FPR2 membrane abundance

**Figure 3.**
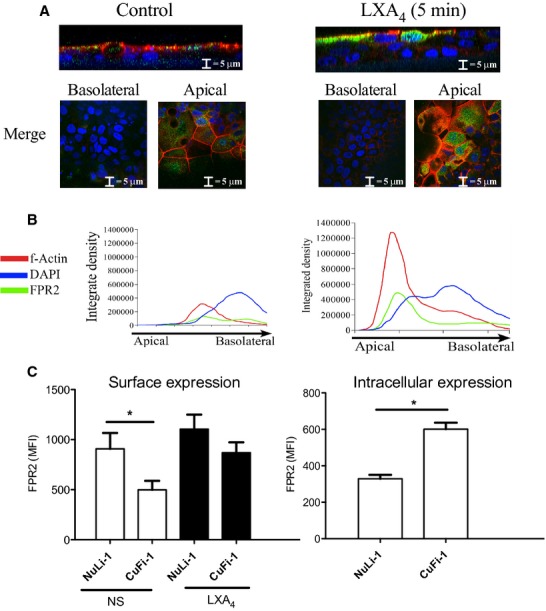
LXA_4_ induced apical membrane ALX/FPR2 localization in CuFi‐1 monolayers. LXA_4_ (1 nmol/L, 15‐min treatment) induced an apical increase of ALX/FPR2 (green) expression (B). Primary rabbit anti‐ALX/FPR2 antibody and secondary Alexa‐Fluor 488 anti‐rabbit were used to label the ALX/FPR2 receptor. Localization of the receptor at the apical surface is shown in the merged fluorochrome images in yellow (A). Rhodamine‐phalloidin was used to stain f‐actin and DAPI used to stain the nuclei. (C) FACS analysis of surface versus cytosolic localization of the ALX/FPR2 receptor in response to LXA_4_ (1 nmol/L, 15‐min treatment) in Nuli‐1 and CuFi‐1 cells (*n* = 4, *P < 0.05).

The localization of the ALX/FPR2 receptor expression was investigated using immunofluorescence staining on well‐differentiated CuFi‐1 epithelial monolayers (Fig. 3A). Short‐term stimulation of CuFi‐1 monolayers induced an apical increase in the integrated density of the secondary Alexa‐Fluor 488 signal for ALX/FPR2 (Fig. 3B). This apical translocation of the receptor was accompanied by a concentration peak in the F‐Actin signal at the membrane. The apical translocation of the intensity of the ALX/FPR2 and F‐actin signals indicate cytoskeletal reformation in response to LXA_4_ exposure. Expression of the ALX/FPR2 receptor was further investigated by FACS analysis. LXA_4_ treatment increased the surface expression of the receptor in CuFi‐1 cells (*n* = 4, *P* < 0.05, *t*‐test, Fig. 3C). Taken together, these results indicate that the ALX/FPR2 receptor is localized in the apical membrane of airway epithelial cells and that LXA_4_ stimulated trafficking and abundance of the receptor at the cell surface.

### Contribution of amiloride‐sensitive ion transport to ASL height regulation by lipoxin A_4_

**Figure 4.**
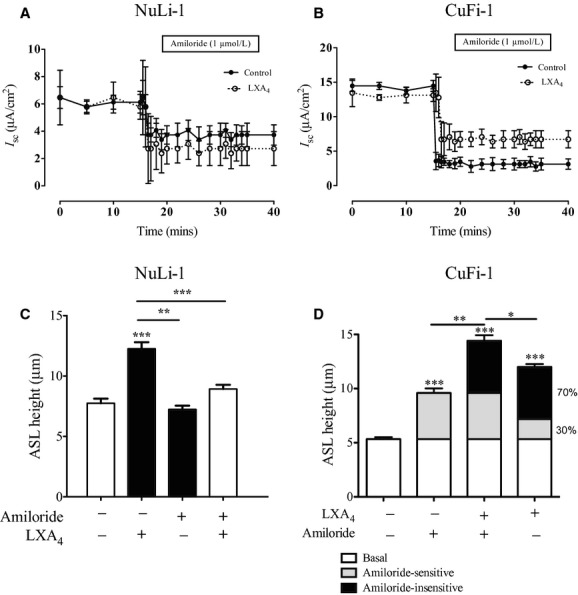
Effect of LXA_4_ on amiloride‐sensitive ion transport and ASL height regulation. Response of the amiloride‐sensitive current to LXA_4_ (1 nmol/L) pretreatment in NuLi‐1 (A) & CuFi‐1 (B) monolayers. Effect of apical amiloride (1 *μ*mol/L, 15 min treatment) application on the LXA_4_ mediated increase in ASL height in NuLi‐1 (C) CuFi‐1 (D) monolayers (*P < 0.05, **P < 0.01, ***P < 0.001).

The role of ENaC in the response of ASLh to LXA_4_ was tested using the ENaC channel blocker amiloride. Apical amiloride exposure induced a mean decrease of the transepithelial short‐circuit current (I_SC_) by 11.28 ± 1.28 *μ*A/cm^2^ (from 14.40 ± 2.40 to 3.10 ± 0.56 *μ*A/cm^2^ *n* = 9, *P* < 0.05) in CuFi‐1 monolayers compared to 1.96 ± 0.28 *μ*A/cm^2^ (from 6.05 ± 0.43 to 4.43 ± 0.47 *μ*A/cm^2^ *n* = 4, *P* < 0.05) in NuLi‐1 monolayers (Fig. 4B). Thus, ENaC activity accounts for a major component of the total transepithelial current in CF cultures (approx. 78%) and relatively little in non‐CF cultures (33%). The increased amplitude of the amiloride‐sensitive I_SC_ response in CuFi‐1 monolayers is consistent with a major role for Na^+^ absorption through ENaC in generating the transepithelial current following the loss of a contribution by CFTR (Chen et al. 2010).

Acute LXA_4_ application to the apical or basolateral chambers of the Ussing chambers did not result in any change of the transepithelial current. In NuLi‐1 cells, LXA_4_ did not affect the amiloride‐sensitive current compared from a basal level of 1.96 ± 0.28 *μ*A/cm^2^ to LXA_4_ treatment of 2.05 ± 0.68 *μ*A/cm^2^ (*n* = 8, Fig. 4A). However, pretreatment with apical LXA_4_ reduced the amiloride‐sensitive current in CuFi‐1 monolayers from 10.45±1.26 *μ*A/cm^2^ to 6.39 ± 0.58 *μ*A/cm^2^ (*n* = 7, *P* < 0.05, Fig. 4B).

The significance of inhibition of Na^+^ transport by LXA_4_ was investigated on ASLh dynamics. In NuLi‐1 cells, ENaC inhibition with amiloride did not affect the ASLh (Fig. 4C). This result highlights that sodium absorption does not contribute significantly to the basal ASLh in non‐CF epithelia and is also consistent with the low amiloride‐sensitive currents observed under short‐circuit current conditions (Fig. 4A). In contrast, in CuFi‐1 cells, amiloride significantly increased the ASLh from 5.3 ± 0.2 *μ*m (*n* = 7) to 9.6 ± 0.3 *μ*m (*n* = 6, *P* < 0.01, Fig. 4D). These findings highlight the contribution of the enhanced Na^+^ absorption to reduce the ASLh in the CF epithelium. The contribution of Na^+^ absorption via ENaC to the ASLh increase is shown in Figure 4D (gray bars).

LXA_4_ independently stimulated a larger ASLh increase than amiloride in CuFi‐1 monolayers. Therefore, lipoxin must affect two separate (amiloride‐sensitive and amiloride‐insensitive) ion transporter systems to effect an increase in ASLh. When added in combination, amiloride and LXA_4_ produced additive effects on ASLh and the contribution to the ASLh of the amiloride‐insensitive transporter can be deduced when compared to LXA_4_ and amiloride alone and is highlighted in Figure 4D (black bars). Assuming that the amiloride‐sensitive and amiloride‐insensitive ion transporter effects are independent, their respective contributions to the ASLh generation can be calculated after LXA_4_ exposure. By this analysis, the amiloride‐sensitive and amiloride‐insensitive currents contributed 30% and 70%, respectively, to the LXA_4_‐mediated ASL height increase in the CF cultures (Fig. 4D). Taken together, the results identify that ENaC inhibition contributed 30% to the ASLh generation induced by LXA_4_, while the remaining 70% was due to the regulation of other ion channels, likely to be involved in Cl^−^ secretion as shown below.

### Lipoxin A_4_ effects on bumetanide‐sensitive ion transport and ASL height

**Figure 5.**
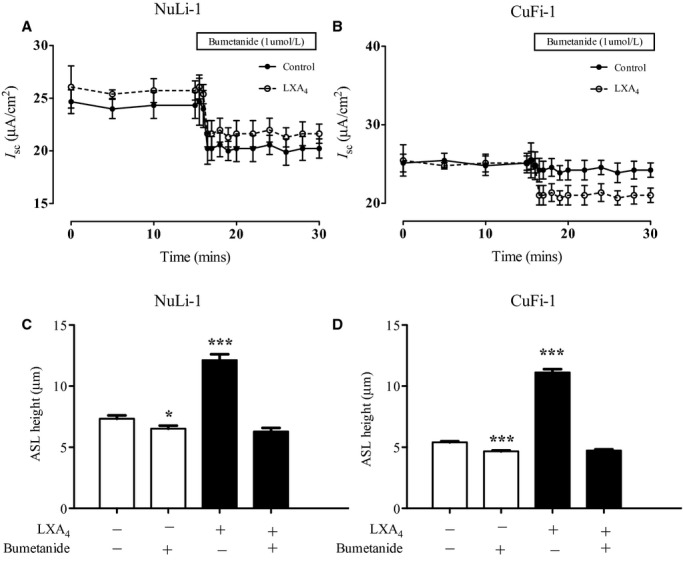
Effect of LXA_4_ on bumetanide‐sensitive ion transport and ASL height. (A) The response of bumetanide‐sensitive current to LXA_4_ (1 nmol/L) pretreatment in NuLi‐1 (A) & CuFi‐1 (B) monolayers. Effect of apical bumetanide (1 *μ*mol/L, 15‐min treatment) application on the LXA_4_ mediated ASL height increase in NuLi‐1 (C) and CuFi‐1 (D) monolayers.

The two major conducting pathways involved in airway transepithelial ion transport are Na^+^ absorption via ENaC and Cl^−^ secretion via CFTR and Ca^2+^ ‐activated channels (CaCC). Short‐circuit current experiments with LXA_4_ pretreatment (1 nmol/L) of NuLi‐1 monolayers for 15 min increased the bumetanide‐sensitive current from 2.90 ± 0.21 *μ*A/cm^2^ (*n* = 27) to 4.36 ± 0.45 *μ*A/cm^2^ (*n* = 6, *P* < 0.05, Fig. 5A). However, in CuFi‐1 monolayers, the basal bumetanide‐sensitive current was almost absent (0.49 ± 1.9 *μ*A/cm^2^, *n* = 9), whereas after 15 min pretreatment with LXA_4_ (1 nmol/L) the transepithelial Cl^−^ current increases to 3.73 ± 0.98 *μ*A/cm^2^ (*n* = 3, *P* < 0.05, Fig. 5B).

Exposure to basolateral bumetanide decreased the steady‐state ASLh in NuLi‐1 epithelium from 7.3 ± 0.3 µm (*n* = 6) to 6.5 ± 0.3 µm (*n* = 10, *P* < 0.001). Similarly, the baseline ASLh in CuFi‐1 monolayers was significantly decreased by bumetanide from 5.5 ± 0.1 µm (*n* = 29) to 4.8 ± 0.1 µm (*n* = 24, *P* < 0.001). Bumetanide abolished the ASLh increase induced by LXA_4_ in NuLi‐1 from 12.13 ± 0.48 µm (*n* = 11) to a height of 6.29 ± 0.29 µm (*n* = 8, *P* < 0.001, Fig. 5C). Similarly, the LXA_4_‐mediated ASLh increase in CuFi‐1 was abolished by bumetanide from a stimulated height of 11.12 ± 0.27 µm (*n* = 30) to a height of 4.73 ± 0.09 µm (*n* = 23, *P* < 0.001, Fig. 5D) indicating a major contribution of Cl^−^ transepithelial transport to the stimulation of ASLh by LXA_4_. We have already shown that LXA4 activates CaCC channels in CuFi‐3 cells and CF primary bronchial cell cultures (Bonnans et al. 2003; Verriere et al. 2012). We could find no effect of the CFTR inhibitor Inh172 on Isc after LXA4 treatment (data not shown). Thus, there is no evidence from specific CFTR inhibitor studies that LXA4 stimulates deltaPhe508CFTR or its trafficking to the membrane.

## Discussion

Cystic fibrosis (CF) is a lethal genetic disorder resulting from a mutation of the CFTR gene coding for a Cl^−^ channel normally localized in the apical membrane of epithelial cells (Riordan et al. 1989). CF is characterized by dehydration and reduction of the airway surface liquid layer. This results in an impaired mucociliary clearance of pathogens from the lung, chronic pulmonary infection, and inflammation. Clearance of airway secretions has been a first‐line therapy for CF patients (Pisi and Chetta 2009). CFTR functions as a cAMP‐activated Cl^−^ channel and as a regulator of other ion channels, such as inhibition of ENaC activity. In CF, the absence of CFTR repression of ENaC activity results in increased absorption of Na^+^ and secondary dehydration of the ASL layer (Stutts et al. 1995; Kunzelmann et al. 1997; Kunzelmann 2003). ASL dehydration results in chronic bacterial infection, persistent inflammation, and progressive lung destruction. The majority of previous studies on ASL dynamics have been performed using differentiated primary human epithelial cells. Our approach using well‐differentiated NuLi‐1 and CuFi‐1 cell lines grown under thin film conditions has shown to be a robust and reproducible method of measuring a dynamic ASL and thus provides a new model system for CF ion transport and cell signaling and supplement human primary tissue studies. ASL stabilization kinetics demonstrated a more rapid attainment of plateau values in the CF model when compared to the non‐CF monolayers. The mean plateau value of the stabilized ASLh was reduced in the CF cell model which is consistent with a diminished ASLh in CF airways (Boucher 2004a).

Short‐term exposure to very low physiological (nanomolar) concentrations of LXA_4_ induced an increase in ASLh in CF and non‐CF bronchial epithelial cell lines. This result is important for assessing a therapeutic role for lipoxins in CF and is consistent with our previous publication showing that LXA_4_ at much higher concentrations (100 nmol/L) also increased ASLh in airway epithelial cells (Verriere et al. 2012). Here, we show a novel effect for LXA_4_ in inhibiting ENaC activity in addition to its pro‐secretory action (Verriere et al. 2012) and resolution of inflammation (Serhan and Savill 2005). Our results contrast with a recent report indicating that LXA_4_ used at 100 nmol/L stimulated ENaC expression in A549 alveolar cells, whereas LXA_4_ at lower concentrations of 1 and 10 nmol/L failed to affect ENaC (Wang et al. 2013). However, A549 cells may not be a good model to study physiological responses to lipoxin in airway epithelia as it has been previously reported that this alveolar cell line does not express the ALX/FPR2 receptor (Bonnans et al. 2006), whereas the bronchial epithelial cells used in our study show regulated expression of the specific receptor for LXA4 (Buchanan et al. 2013).

The stimulatory effect of LXA_4_ on ASLh was maintained for over 24 h in CF epithelium. Although LXA_4_ is rapidly metabolized (Serhan and Romano 1995; Clish et al. 2000), LXA_4_ might induce long‐lasting genomic effects following the activation of its ALX/FPR2 receptor such as expression of ion transporter and tight junction proteins. The lasting positive effect on airway hydration in CF may provide a novel route in correcting the deficient ASL dynamics in CF airway disease.

ASL depth is tightly regulated by the net transport of Na^+^ absorption and Cl^−^ secretion (Matsui et al. 1998, 2000; Tarran and Boucher 2002). The lining of the airways is characterized as mainly a Cl^−^ secretory epithelium. Activation of Cl^−^ channels in the apical membrane in the airway epithelium leads to efflux of Cl^−^ into the lumen. This electrogenic Cl^−^ efflux provides the potential to drive Na^+^ and water across the epithelial tight junctions to provide net transepithelial salt and water secretion into the lumen. To maintain the chemical gradient and the membrane potential, both under resting conditions and during Cl^−^ secretion, Na^+^ and K^+^ exits across the basolateral membrane via the Na^+^/K^+^ ATPase and basolateral potassium channels, respectively (Jensen et al., 2001). The channels, transporters, and pumps in all secretory epithelia operate in concert to achieve net transepithelial ion transport. Therefore, inhibition of any one of these pathways attenuates the transepithelial transport of Cl^−^, and consequently decreases the rate of salt and water secretion (Jensen et al., 2001). Our study demonstrates that lipoxin affects both secretion and absorption in airway epithelium. In non‐CF bronchial epithelium, lipoxin increases ASL height by inhibiting ENaC to decrease Na^+^ absorption and by activating CFTR to enhance Cl‐secretion. In CF bronchial epithelium, lipoxin restores ASL height mainly by inhibiting ENaC in the absence of CFTR activity.

The findings strengthen evidence on the physiological role of ENaC in maintaining the lung liquid balance as demonstrated in mice in which the *α*‐ENaC gene was inactivated by homologous recombination (Hummler et al., 1996). Neonatal mice developed respiratory distress and died prematurely at 40 h from failure to clear the lungs of fluid. Patients with pseudohypoaldosteronism who exhibit functional loss of ENaC have an excess ASL due to reduced Na^+^ absorption (Kerem et al., 1999). In this group of patients, the accelerated mucus transport is probably due to increased ASL as demonstrated by Tarran et al. (2001).

In CF, changes in Na^+^ absorption are secondary to absence of the CFTR channel (Chen et al. 2010) due to release of inhibition from CFTR (Stutts et al. 1995; Kunzelmann et al. 1997). Acute addition of LXA_4_ did not affect the Isc. We also tested the effects at 15, 30, 45, and 60 min and there was no effect on Isc at all time frames. This result is not surprising given the opposite effects of LXA_4_ to inhibit Na^+^ absorption (decreases Isc) and stimulate Cl^−^ secretion (increases Isc) which may cancel out any net change in Isc. The data from short‐circuit current and ASLh measurements (under open‐circuit conditions) are internally consistent. In the non‐CF cultures, LXA_4_ has little effect on both amiloride‐sensitive I_SC_ and ASLh. In contrast, in the CF cultures, the amiloride‐sensitive I_SC_ accounts for ~80% of the total I_SC_ and this is reflected in the large effects of amiloride on ASLh.

We demonstrated that LXA_4_ stimulates a bumetanide‐sensitive Cl^−^ current coupled to ASLh regulation in both normal and CF airway epithelia. This result is consistent with our previous report showing that LXA_4_ stimulated a calcium‐dependant Cl^−^ transport (Bonnans et al. 2003; Verriere et al. 2012). Thus, LXA_4_ increases the ASL height in CF epithelium by two complementary pathways through inhibiting Na^+^ hyperabsorption via ENaC and stimulating Cl^−^ secretion via CaCC channels. The dual effect of LXA_4_ on ENaC and CaCC strengthens the relevance of our findings for a possible therapeutic effect of LXA_4_ to reverse all of the ion transport dysfunction in CF airway epithelium to restore a physiological ASL height.

LXA_4_ binds to the ALX/FPR2 receptor to elicit its effects in a variety of cells (Serhan et al. 1984; Serhan 1997). Here, we report for the first time the presence of ALX/FPR2 expression in CF airway epithelial cells. Although the ALX/FPR2 receptor protein is expressed in the cytoplasm, it appears to be predominately expressed at the apical membrane of airway epithelial monolayers. A role for LXA_4_ on cytoskeleton reorganization has been shown in monocytes and macrophages (Maderna et al. 2002) and is consistent with our findings that the ALX/FPR2 receptor is translocated to the apical surface upon exposure to LXA_4_.

Donabedian and Gallin (1981) showed that there was a transient agonist‐induced decrease in the number of FPR‐binding sites in response to an agonist, and that these binding sites could return to the cell surface if the cells were kept at 37°C (Donabedian and Gallin 1981). The study demonstrated a recycling pool of formyl peptide receptors. Jesaitis and colleagues initiated studies of formyl peptide receptor interaction with the cytoskeleton, and found that a receptor–cytoskeleton complex was formed before receptor internalization which was resistant to Triton X‐100 (Jesaitis et al. 1984, 1985). The findings revealed that FPRs interact with intracellular cytoskeletal proteins which affected the binding properties of the receptor. Further studies on recombinant FPR1 demonstrated agonist‐induced actin polymerization and chemotaxis in transfected HL‐60 cells (Prossnitz et al. 1993). LXA_4_ induces signals that regulate BLT1, production of chemokines, cytokines (e.g., TNF) growth factor receptors (e.g., VEGF) in human leukocytes, and mucosal epithelial cells, each contributing to regulate the resolution of the inflammatory cycle (Serhan and Savill 2005). The level of control by LXA_4_ of key processes relevant to acute inflammation raises the question of how LXA_4_ binding to FPR2/ALX may translate into anti‐inflammatory and pro‐resolving properties. FPR2/ALX is phosphorylated in an agonist‐dependent manner, but little is known about the kinases involved and the determinants responsible for its internalization have not yet been recognized. Phosphorylation of these receptors is known to affect their internalization; it would be interesting to determine if constitutive phosphorylation of FPR2/ALX is related to its cell surface expression pattern.

The increase in ASLh induced by LXA_4_ through the inhibition of Na^+^ hyperabsorption and stimulation of Cl^−^ secretion may prove to be of therapeutic value in CF by correcting normal ion transport, restoring ASLh and hydration, and improving mucociliary clearance. The effect of LXA_4_ in CF cultures raises interesting implications for its potential use in a clinical setting. Currently, drug therapy for CF is limited to the G551D mutation with the potentiator VX‐770 which is restricted to a small percentage (4%) of CF patients. The beneficial effects of LXA_4_ on ASL dynamics may not have such a restricted genotypic use as it corrects the general NaCl transport dysfunction and may complement existing therapies to enhance airway mucociliary clearance and prevent ASL depletion.

## Conflict of Interest

None declared.
